# Gastrointestinal Tract Leak: Is It One Entity or Spectrum of Conditions?

**DOI:** 10.7759/cureus.10458

**Published:** 2020-09-14

**Authors:** Faiz Tuma

**Affiliations:** 1 General Surgery, Central Michigan University College of Medicine, Saginaw, USA

**Keywords:** anastomosis leak, gastrointestinal leak, abdominal sepsis, intra-abdominal collection, contained leak, intraperitoneal free air, intraperitoneal free fluid, gastrointestinal perforation

## Abstract

Gastrointestinal (GI) leak is a well-known and catastrophic surgical complication. Its impact on patients, surgeons, and the healthcare system is tremendous. Efforts to constraint the occurrence and consequences of GI leak contributed to better assessment and management planning, especially with advanced technology. Detail information about the problem extent and new management options became available and effective for specific categories. Therefore, a full and accurate assessment and understanding of the disease presentation assists in choosing the appropriate management plan.

The pathophysiologic process encompasses a severe inflammatory process with a superimposed infection inside sterile body tissue and cavities initiated by contaminated GI leaked content. The extent of the morbidity resulting from GI perforation and leak is variable and may not be predictable. Leak might not be the same in every case. Patients with GI leak present at variable severity depending on several factors. Accordingly, management should be individualized to target the underlying pathophysiology and the extent of the complication. Operative intervention and repair of the perforation site surgically or endoscopically are the standard of care frequently used. However, it may not always be needed. In this article, a practical review of the diversity and underlying pathologies of GI leak will be presented to inform case-specific management plans.

## Introduction and background

A leak of gastrointestinal (GI) content to the thoracic, abdominal, and pelvic cavities or retroperitoneal space is one of the most serious complications of GI diseases or surgical procedures. It is a common surgical complication and entity. The impact on health care and expenses is significant. Therefore, understanding the condition in-depth may assist in improving our management planning and treatment outcomes. 

The extent of the morbidity resulting from GI perforation and leak is variable, may not be predictable, and can lead to death [1]. Leaks are associated with a wide spectrum of complications, such as local infection, sepsis, and multiple organ system failure, in addition to prolonged hospitalization and decreased quality of life. Studies have also shown that leaks are associated with a reduced life expectancy [2,3]. There are multiple factors that determine the severity and extent of the complications.

Patients with GI leak present at variable stages of severity depending on several factors. Hence, treatment varies according to the presentation in addition to the underlying condition. It is therefore important to explore the diversity of presentation and the underlying mechanisms of each to provide better management to each category or presentation level. In this article, the diversity of the presentation and underlying pathophysiology will be reviewed for a better understanding of the condition to provide enhanced care.

## Review

Pathophysiology

GI leak starts after perforation of the wall, either as a consequent to disease, trauma, or surgical intervention [4,5]. The pathophysiologic process starts with contaminated GI content escaping to the sterile body cavities. This initiates a severe inflammatory process with a superimposed infection. The process might spread over large areas of the body cavity causing more severe local and systemic responses. The peritoneal and pleuritic membranes are highly absorptive for the inflammatory and chemical toxins that are produced by the inflammatory response to the leaked material. Therefore, the main factor in treating this condition is to stop the leak or control its spread. 

Stopping GI leak can be done in different ways. Surgical intervention and repairing the perforation site surgically or endoscopically is the common treatment and standard of care. However, at other times, the leak stops spontaneously. Or, in other words, the body reaction seals the perforation site by the adherence of surrounding tissue to the perforation site. The omentum is known to be the first tissue that moves and adheres to the affected site. Other tissue like the small bowel loops, the abdominal wall, and the liver can participate in the sealing process. Therefore, the leak process may be short, intermittent, or continuous depending on many factors. The extent and progress of the leak process will determine the severity and extent of the complications. Therefore, determining whether the leak process is active and continuous or not is crucial in deciding the management approach and expecting the consequent complications. 

At the time of diagnosis, the leak process has already started, the clinical manifestations are developing, and some of the complications have already happened, e.g. fluid collection and superimposed infection. The body sealing mechanisms of the perforation site have already begun and might be completed. Therefore, the question comes of how important to specifically confirm whether the process of the leak is active and ongoing or not [6]. Theoretically, the difference is very important. It affects the management plan and possibly the resulting complications. Practically, this difference might not be that important because the consequence of leak is developing and have to be controlled and reversed.

Management principles

The current management practice focuses on identifying the presence of perforation and leak. Generally, this necessitates surgical intervention for two main reasons: the primary one is to identify and seal the leak, and the secondary one is to ameliorate the consequences of the leak, such as the severe inflammation and infection that is done by washing and draining the leaked collection. This management has been the current practice for decades. The rationale for this practice is that the perforation, leak, and consequent complications are potentially fatal if not aggressively treated by surgical intervention with supportive medical treatment to stop and reverse the leak and its complications [7-10].

The experienced surgeons that have taken care of many of these situations realize that by the time surgery is done the leak might have already been sealed. Sealing can take place by other organs and tissues like the omentum, loops of bowel, and the abdominal wall. The consequent complications of fluid collections are evident and are usually addressed during the surgery time by sufficient drainage and washing. The perforation site is often not easy to identify without careful and tedious inspection and some dissection of the acute fibrinous adhesions. This dissection, on many occasions, necessitates unsealing of the perforation. Then, finally, the sealing has to be done in a secure and optimum surgical fashion that may include resection of the perforated segment [8,11-13].

The operating surgeon takes no, or extremely low, chance of re-leaking after the surgical intervention. Re-leaking will not be tolerated by the patient, the surgeon, or the caring team. The patient can quickly and severely deteriorate after re-leaking. Most of the physiologic reserve has been used. The defense mechanism is at its peak. Prolonging the exposure to the severe inflammatory and infectious process produced by the re-leaked GI content causes poor prognosis.

Having the patient already under anesthesia with good field exposure allows for the best possible surgical sealing and the least possible risk of further complications. This is not a questionable approach once the decision is made to take the patient to surgery [14]. There might be some exception to the approach due to patients or disease factors. But if the decision to operate has not been made or the surgical approach has not been selected yet, the condition can be approached differently depending on multiple factors. The surgical approach is still the gold standard management. However, other invasive or conservative approaches might be appropriate if the patient's condition dictates or allows. One of the very important factors is the patient's stability and the presence of minimal symptoms or complications [15]. In some of these occasions of stable asymptomatic patients, the pathology is identified incidentally on imaging. Free air or extraluminal fluid collection is found and considered as evidence of GI perforation and content leak [16]. At some stages of practice, these findings on simple imaging are considered sufficient to operate. This might still be valid. However, the practice now is to obtain further information of where the perforation could be and if the leak is continuous or is sealed. Knowing the perforation site helps in planning for the appropriate surgical approach.

Presentation diversity

A continuous leak and sealed leak are relatively different situations. Knowing which one of them is happening helps in deciding when or whether to operate. On occasions, a perforation or leak is only discovered after the event has resolved. There are other occasions where the perforation's consequences are clinically not evident. Again, the current practice is to investigate further to characterize the possible perforation and assess the leak consequences. Further imaging with GI luminal contrast is the traditional investigation. No leak of the contrast outside the GI is reassuring. If the imaging shows a limited consequence as in a CT with a minimal collection around the bowel, this provides reassurance and may defer the need for surgical intervention. A common example is the limited sigmoid diverticulitis perforation or Hinchey class II diverticulitis. With this disease, we had long experience and treatment strategies to choose confidently not to operate. But in other diseases, the experience might not be that developed or matured even though it is being reviewed and evaluated. A common example is the sealed cecal perforation. 

So, why do we treat diverticulitis with small perforation (Hinchey class I and II) non-operatively, while a free intra-abdominal air pocket of perforated viscus is enough for urgent or emergent surgical intervention? Are the two situations totally different? Or the disease processes are different? Or our knowledge is high in one situation and low in the other situation? Or something else that we are unaware of and have not studied well yet.

There are a few factors that need a thoughtful review and assessment to better understand why we do what we do.

1. Confirming the underlying pathological process. It helps in planning the management and choosing the appropriate approach. Perforated diverticulitis can be identified and confirmed with imaging much simpler than perforated peptic ulcer disease.

2. Disease behavior. Esophageal perforation behaves much more aggressively than perforated appendicitis.

3. Surgical experience and history. Our knowledge and experience of certain diseases are more than others. An example is sigmoid diverticulitis perforation compared with jejunal diverticular perforation [17]. Knowing the disease pattern well may facilitate choosing the non-rushing treatment approach.

4. Site of perforation. Retroperitoneal duodenal perforation from endoscopic retrograde cholangiopancreatography (ERCP) intervention is different and limited than free Meckel’s diverticulum perforation [18].

5. Extent of the perforation. Bowel perforation from abdominal trauma is more extensive than a polypectomy site colonic perforation [19]. 

Here the argument comes of what if the leak has stopped and the perforation has been sealed naturally? Is this not enough to rely on? and why? To answer these questions and finalize a treatment plan, two components of the surgical problem have to be addressed. The first one is the consequences of the GI unsterile content leak and the resulting peritonitis. A significant amount of leaked content and collection will induce severe peritonitis complicated by serious infection. The entire abdominal cavity environment becomes severely inflamed and infected, leading to tissue damage and friability. A later attempt of operating or repair of bowel will not be technically possible due to the severe tissue inflammation and friability. The exception of this situation is the controlled leak where the leaking contents are drained to outside the abdominal cavity by drains [20]. The second component is the leak process, whether it is continuous or active or it has stopped by the natural body defense mechanism at the time of diagnosis. As discussed above, signs of continuing leaks indicate the need for urgent surgical treatment. For a more cautious approach, the absence of evidence of sealed perforation is an indication for urgent surgical repair. 

This leads to the conclusion that a significant amount of free GI content from a perforation needs urgent surgical intervention. A GI perforation from which a leak cannot be excluded is also an indication for urgent surgical intervention. Other than that, cases have to be taken individually case by case to consider all the other factors [21]. Leaning toward operative is a safe clinical treatment approach to GI leak in general.

## Conclusions

GI leak presents as a spectrum of severity or conditions. Assessment and management planning have to be tailored on a case-by-case basis. The specific goal of management should direct the care plan. Continuous re-assessment and hence plan revision are prudent. Operative management is always safer but is not always necessary.
